# Health service readiness for implementing the National Sickle Cell Anaemia Elimination Mission in Chamarajanagar District, Karnataka, India: a cross-sectional mixed-methods protocol

**DOI:** 10.1136/bmjph-2026-005317

**Published:** 2026-09-17

**Authors:** Anandhu K Ramesan, Tanya Seshadri, Manashri Bhuyar, Deepa Bhat

**Affiliations:** 1 Anatomy, JSS Medical College, JSS Academy of Higher Education and Research, Mysuru, Karnataka, India; 2 Center of Adivasi Health, Institute of Public Health Bengaluru, Bengaluru, Karnataka, India; 3 Institute of Public Health Bengaluru, Bengaluru, Karnataka, India

**Keywords:** Community Health, Public Health, Health Services Accessibility

## Abstract

**Introduction:**

Sickle cell disease (SCD) remains a major public health challenge in India, disproportionately affecting tribal populations. In 2023, the Government of India launched the National Sickle Cell Anaemia Elimination Mission (NSCAEM) to strengthen screening, diagnosis and long-term care. However, evidence on the readiness of health facilities to implement SCD-specific services under NSCAEM is limited, particularly in tribal and resource-constrained settings. This protocol outlines a systematic assessment of health-system readiness for NSCAEM implementation in a predominantly tribal district of Karnataka.

**Methods and analysis:**

This study will adopt a cross-sectional, facility-based, mixed-methods design in Chamarajanagar District, Karnataka, conducted between September 2024 and March 2027. All public health facilities involved in SCD screening, diagnosis, referral or management, across primary, secondary and tertiary levels, will be included. Quantitative data will be collected using an adapted WHO Service Availability and Readiness Assessment toolkit, incorporating indicators for SCD diagnostics, medicines, emergency care, counselling and referral systems. The adapted toolkit was developed through expert consultation and piloted at four facilities. Readiness will be assessed using tracer indicators and summarised through domain-specific scores and a composite Service Readiness Index, with a planned sensitivity analysis comparing equal-weighted and expert-weighted domain scores. Qualitative data will be collected through approximately 15–20 key informant interviews and a stakeholder validation workshop to contextualise quantitative findings and explore implementation challenges. Descriptive and comparative analyses will be undertaken, with triangulation of quantitative and qualitative results.

**Ethics and dissemination:**

Ethical approval for this study has been obtained from the Institutional Ethics Committee of JSS Academy of Higher Education and Research, Mysuru, Karnataka (Reference: JSS/MC/PG/217/2024-25). Findings will be disseminated through policy briefs to district and state health authorities, stakeholder workshops, community forums and peer-reviewed open-access publications.

**WHAT IS ALREADY KNOWN ON THIS TOPIC**

- Readiness assessments such as the WHO Service Availability and Readiness Assessment (SARA) framework are widely used to evaluate health-system capacity across different programmes.
- Evidence on health-system readiness for sickle cell disease (SCD) services in India, particularly in tribal regions, remains limited.
- The National Sickle Cell Anaemia Elimination Mission (NSCAEM) prioritises screening and care; however, district-level implementation readiness has not been systematically evaluated.

**WHAT THIS STUDY ADDS**

- This is the first protocol to adapt the WHO SARA toolkit specifically for SCD services within the context of NSCAEM in India, with an explicit account of the tool’s development, piloting and an empirical reliability check.
- The study introduces SCD-specific readiness indicators, including diagnostics, emergency care, counselling and continuity-of-care systems, and reports a pre-specified sensitivity analysis for the composite readiness index.
- Findings will generate actionable readiness scores and implementation insights to inform resource allocation and programme planning in high-burden tribal districts.

**HOW THIS STUDY MIGHT AFFECT RESEARCH, PRACTICE OR POLICY**

- This study provides a replicable framework for assessing readiness for genetic-disease programmes in resource-constrained settings.
- It supports policy decisions to strengthen SCD services at the primary, secondary and tertiary levels.
- It offers an evidence base to refine NSCAEM implementation nationally, while clearly delineating readiness from downstream clinical outcomes.

**Strengths and limitations of this study**

- This study applies a standardised, replicable framework to assess sickle cell disease-specific service readiness at the district level, integrating quantitative and qualitative methods.
- The adapted toolkit was refined through expert consultation and field piloting, and its measurement consistency was checked through an independent, blinded re-assessment of a random subsample of facilities during data collection.
- The cross-sectional design captures readiness at a single time point and does not assess quality of care, service utilisation or patient outcomes.
- The assessment is restricted to public-sector facilities and to the health-system level; private-sector provision and community/patient perspectives are outside its scope.
- Findings are specific to a single tribal district and should be generalised to other National Sickle Cell Anaemia Elimination Mission-implementing districts with caution.

## Introduction

 Sickle cell disease (SCD) is an inherited haemoglobinopathy that has emerged as a significant global public health concern due to its substantial morbidity and mortality burden.^1,2^ International agencies, including the United Nations and the WHO, recognise SCD as a priority condition requiring coordinated global action.^3^ Each year, an estimated 330 000 infants are born with SCD worldwide, the majority in sub-Saharan Africa.^2^ The Global Burden of Disease 2021 analysis reported a 41% increase in global prevalence between 2000 and 2021, from 5.46 million to 7.74 million people living with the condition. SCD is among the leading causes of death in children under 5 years of age,^4^ although this burden is likely underestimated; in high-prevalence regions, underdiagnosis and preventable complications such as infection and severe anaemia may contribute to childhood mortality approaching 90% in some settings.^5^

In India, SCD represents a significant and continuing public health challenge, particularly among scheduled tribe communities across the central, western and southern states.^6^ A systematic review estimates the national prevalence of SCD at 1.17% and of sickle cell trait (SCT) at 5.9%, with considerable geographic variation; the highest burden is reported in the central and eastern regions, while SCT prevalence is notably high in the south.^7^ Although some Indian studies describe comparatively milder phenotypes than those reported in African contexts, attributed to genetic and environmental factors and higher fetal haemoglobin levels,^8^ severe vaso-occlusive crises, life-threatening complications and frequent hospitalisations remain common.^9^ The assumption that SCD in India is uniformly mild is therefore misleading, and the disease continues to contribute to preventable morbidity and mortality.^10^

Recognising this burden, the Government of India launched the National Sickle Cell Anaemia Elimination Mission (NSCAEM) in 2023.^11,12^ As a newly introduced national programme being integrated into an already complex health system with multiple ongoing national health initiatives, assessing health-system readiness is essential to ensure effective implementation and avoid service fragmentation. The mission aims to eliminate SCD as a public health problem by 2047 through strategies that include awareness generation, universal screening, early diagnosis, counselling and strengthened clinical management, with a particular emphasis on tribal and high-prevalence areas.^11,12^ Despite the rapid expansion of screening activities, gaps persist. Access to confirmatory diagnostics, comprehensive follow-up and disease-specific care remains limited in rural and remote settings, where tribal communities often face structural barriers to health services.^6,13,14^ Coexisting genetic conditions such as β-thalassaemia and glucose-6-phosphate dehydrogenase deficiency further complicate clinical management.^15^

These challenges must be understood within broader systemic constraints affecting rural healthcare delivery in India.^16^ Weak laboratory infrastructure, shortages of trained personnel, limited emergency preparedness at primary-care facilities and fragmented referral systems undermine the timely diagnosis and effective management of SCD.^17,18^ As a result, the benefits of population-wide screening risk being undermined if the health system is insufficiently prepared to provide longitudinal care.

Health-service readiness—the capacity of facilities, providers and systems to deliver essential services—has therefore become central to programmatic success. Consistent with Donabedian’s structure-process-outcome framework,^19^ readiness assessments generate structured information on infrastructure, human resources, diagnostics, medicines and service organisation (structure), enabling health planners to identify bottlenecks and prioritise investments; they do not, by design, capture the quality of care delivered (process) or the health gains achieved (outcome). The WHO Service Availability and Readiness Assessment (SARA) framework provides a standardised, globally used approach for assessing whether facilities can provide essential services across multiple domains.^20^

To our knowledge, no study has systematically assessed health-system readiness for SCD services within the context of NSCAEM. Existing SARA-based assessments in India and other low- and middle-income settings have primarily focused on maternal and child health, infectious diseases and non-communicable diseases, with considerable success in guiding policy and implementation frameworks in these fields, but have not extended to genetic disorders or tribal health contexts.^20–22^ Evidence specific to health-system readiness for SCD care or other genetic disorders is exceptionally scarce.

The present study seeks to address this gap by adapting the WHO SARA framework to incorporate programme-specific and disease-specific indicators relevant to SCD, and by applying this adapted tool in a predominantly tribal district of Karnataka. The focus district, Chamarajanagar, is characterised by geographically remote settlements, several tribal communities and limited healthcare infrastructure. The district also has a recognised SCD burden, making it a relevant setting for a readiness assessment under NSCAEM.^23^ By examining service availability, diagnostic and treatment capacity, workforce readiness and referral preparedness across levels of care, the study aims to generate actionable readiness profiles.

The resulting evidence is expected to inform programme planning, resource allocation and health-system strengthening under NSCAEM, while contributing to the broader understanding of readiness assessment for chronic and genetic conditions in resource-constrained settings. We emphasise, however, that this protocol is designed to characterise structural readiness; it is not designed, and should not be interpreted, to measure clinical outcomes, service quality or community-level care-seeking behaviour, each of which requires dedicated research designs discussed further below.

## Methodology

### Study design, setting and timeline

**Figure 1:**
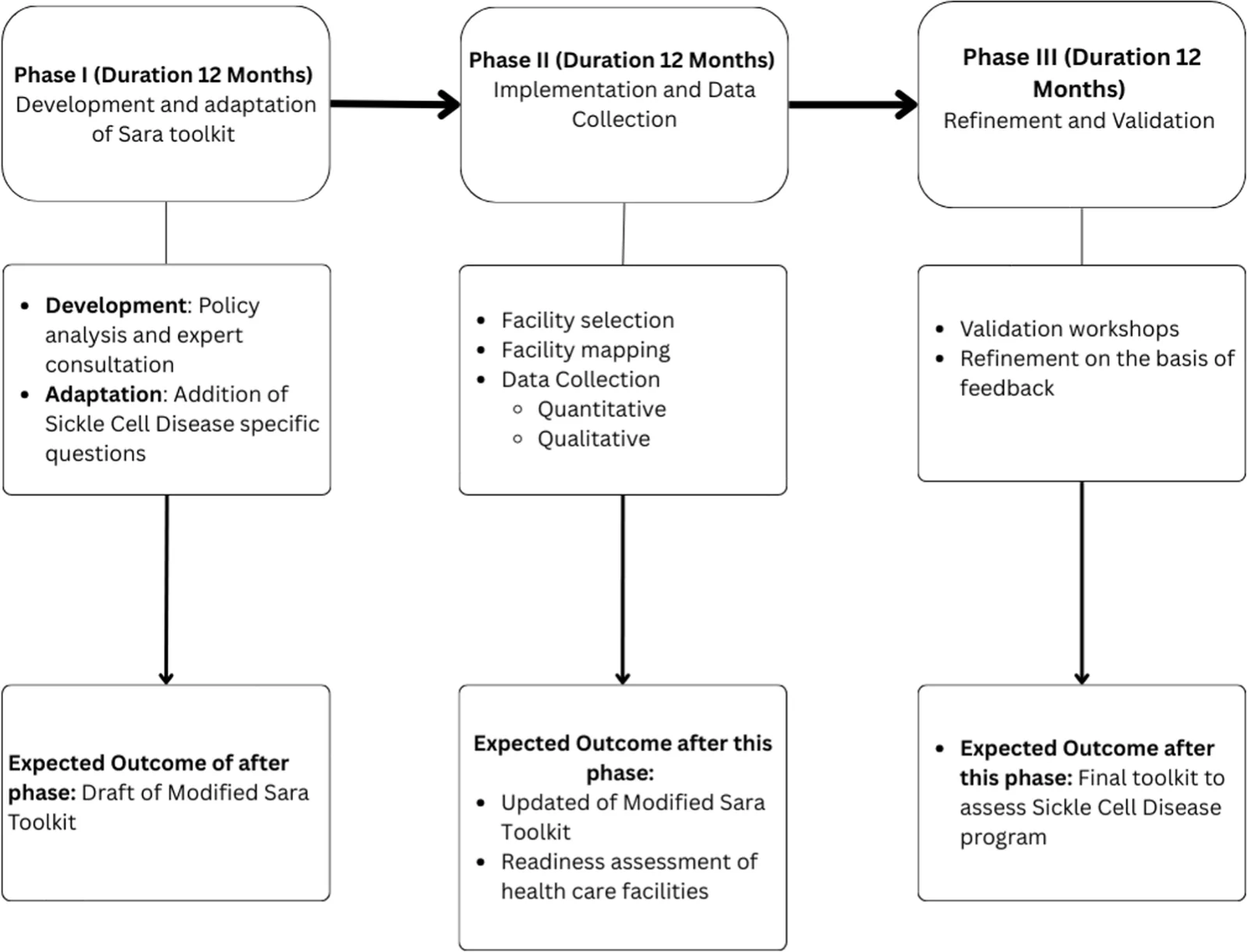
Schematic flow of the study phases. SARA, Service Availability and Readiness Assessment.

This study adopts a cross-sectional, facility-based, mixed-methods design to assess the readiness of public health facilities in Chamarajanagar District, Karnataka, to implement NSCAEM. The assessment is based on an adapted WHO SARA framework incorporating SCD-specific indicators. The study is organised into three sequential phases: toolkit adaptation and validation, field implementation and stakeholder validation (figure 1).

The overall doctoral research programme extends from September 2024 to March 2027. This encompasses protocol development, stakeholder consultation and ethics approval (September 2024 to 2025); toolkit adaptation and piloting (early 2026); facility-based quantitative data collection over a 6-month period, planned from February 2026 to July 2026; qualitative data collection (key informant interviews (KIIs)) following completion of quantitative data analysis, followed by a single stakeholder validation workshop once both the quantitative and qualitative analyses are complete, planned for the second half of 2026; and final analysis, manuscript preparation and dissemination extending into 2027.

The study encompasses all public facilities offering SCD-related services across primary, secondary and tertiary levels, including tribal-focused primary health centres (PHCs), community health centres, taluk hospitals, the sub-divisional hospital, the district hospital and the medical college. A summary of facilities included in the study, their types and numbers, is presented in table 1.

**Table 1:** Public health facilities included in the study, Chamarajanagar District

Facility type	Number of facilities
Primary health centres (PHCs)	24
Community health centres (CHCs)	3
Taluk hospitals (THs)	4
Sub-divisional hospital (SDH)	1
District hospital (DH)	1
Medical college (MC)	1
Total	34

Chamarajanagar District, located in southern Karnataka, has a population exceeding 1 million and a substantial representation of scheduled tribes. The Soliga community comprises the majority of the tribal group, while the Jenu Kuruba, Betta Kuruba and Yerava communities make up smaller proportions. The district consists of five rural taluks: Chamarajanagar, Gundlupet, Kollegala, Hanur and Yelandur.

### Study population and sampling

The sampling strategy was designed to capture the full spectrum of SCD service delivery across levels of care within the public health system. All public facilities involved in SCD screening, diagnosis, referral or management in Chamarajanagar District will be included to maximise representation and avoid selection bias.

At the primary-care level, all 24 tribal-focused PHCs are included, as they are the primary health service providers for most tribal communities. At the secondary level, all three community health centres, four taluk hospitals and the single sub-divisional hospital will be included, together with the district hospital, due to their referral and inpatient care functions. The district medical college and hospital will be included as the tertiary-care facility, given its specialist services, diagnostic capacity and supervisory role. This yields a total of 34 facilities (table 1); the facility inclusion and exclusion criteria are summarised in table 2.

**Table 2:** Facility inclusion and exclusion criteria

Category	Criteria
Facility inclusion criteria	Public health facilities providing SCD-related services under the National Sickle Cell Anaemia Elimination Mission (NSCAEM)
	Located within Chamarajanagar District
	Functional and operational during the study period
Facility exclusion criteria	Private or not-for-profit facilities
	Administrative offices with no clinical service delivery
	Facilities undergoing closure or major renovation preventing assessment

SCD, sickle cell disease.

### Selection of respondents

Within each facility, respondents will be purposively selected based on direct involvement in service delivery and programme implementation. These will typically include the medical officer-in-charge, laboratory technician, staff nurse or Auxiliary Nurse Midwife (ANM), pharmacist and programme or data staff, where available. Where more than one eligible person occupies a role, the individual with the greatest programme experience will be invited to participate.

### Non-participation and replacement procedures

If a facility is temporarily unavailable due to emergencies, staff shortages or administrative constraints, a repeat visit will be scheduled within 2 weeks. If any individual staff member declines to participate, another eligible member in the same role will be approached. No replacement will be made for facilities that decline to participate; such instances will be documented and reported. Since this study aims to include all eligible facilities, no formal sample size calculation is required; conducting a district-wide census enables comprehensive readiness profiling and supports interpretation across levels of care.

### Study phases

#### Phase I: development, adaptation and validation of the assessment toolkit

Phase I comprised a structured, three-step adaptation of the WHO SARA toolkit.

This validation process—literature and policy review, expert consultation and piloting—was directed specifically at the newly introduced SCD/NSCAEM-specific tracer indicators. The core WHO SARA domains and tracer indicators retained without modification (basic amenities, basic equipment, standard precautions for infection prevention and the general diagnostic and medicines modules) were not re-validated in this study, as their content validity, cross-cultural applicability and measurement properties are already established through the WHO SARA instrument’s extensive prior use across multiple countries and health conditions.^20,24–26^ Reliability testing (below) was likewise restricted to the SCD-specific indicators, for which no such precedent exists.

First, the research team conducted a review of existing national and global literature on service readiness for SCD, together with national and state health policy documents—including NSCAEM’s operational guidelines^27^—to ensure alignment with programme expectations and to generate a preliminary list of candidate SCD-specific indicators spanning screening, confirmatory diagnostics, essential medicines, crisis management, counselling and referral.

Second, the preliminary indicator list was reviewed through individual, one-off consultations with five domain experts: two public health researchers, the district tuberculosis officer, who also serves as the district’s designated SCD/NSCAEM nodal officer (this dual role informed judgements on both vertical disease-control operations and SCD-specific programme feasibility) and two medical officers with direct SCD service-delivery experience. Experts were asked to comment on the relevance, comprehensiveness and contextual appropriateness of each candidate indicator, and their feedback was incorporated into indicator wording and response categories. This consultation was conducted as a single round of open-ended discussion rather than a structured Delphi process, and a formal content validity index was not computed at this stage.

Third, the revised toolkit was piloted at four purposively selected facilities—three PHCs and the tertiary medical hospital—to assess item clarity, feasibility, administration time and skip-pattern logic. Piloting resulted in refinements to the phrasing of several diagnostic and crisis-management items and to the observational checklist. Pilot facilities will not be re-included in the main data collection.

As a quality-assurance measure during Phase II, the principal investigator independently and blindly reassessed a random subsample of four facilities using the full assessment toolkit, recording observations before comparing them with the original data collector’s entries for those facilities. Agreement for categorical tracer items will be quantified using Gwet’s AC1^28^ reported alongside Cohen’s kappa^29,30^ for comparability, and agreement for continuous domain scores using the intraclass correlation coefficient (ICC)^31^; these analyses had not yet been performed at the time of this submission and will be reported alongside the main readiness findings. Given the small subsample, these estimates should be interpreted as indicative rather than definitive evidence of measurement consistency.

Based on these inputs, the toolkit was modified to include SCD-specific dimensions, such as the availability of SCD-specific therapeutic drugs (hydroxyurea), diagnostic tools (solubility test, electrophoresis and high-performance liquid chromatography), protocols for managing acute sickle cell crises, blood transfusion capacity and access to genetic counselling services. Readiness will be assessed across the standard SARA domains—basic amenities, basic equipment, infection prevention, diagnostic capacity and essential medicines—as well as SCD-specific indicators for screening, confirmatory diagnostics, clinical management, counselling and referral. The complete list of tracer indicators, scoring approach and domain structure is shown in table 3, with the complete list of SCD-specific tracer indicators provided in online supplemental table S1.

**Table 3:** Overview of SARA modules and sections included in the adapted assessment toolkit

Module	Section	Focus area	Key components
Module 1: service availability	Section 1	Interviewer visit	Facility identification, general information
	Section 2	Staffing	Number and type of staff, availability and deployment
	Section 3	IP and OP beds	Inpatient and outpatient bed capacity, utilisation
Module 2: service readiness	Section 4	Infrastructure	Communication, transport, power supply, basic client amenities, infection control, processing of equipment for reuse, healthcare waste management, supervision, basic equipment, infection control precautions
	Section 5	Available services	Antenatal: services, guidelines, trainings Obstetrics and newborn: immediate and complicated newborn care, guidelines, trainings, equipment, medicines Immunisation: facility and outreach services, guidelines, training, equipment, refrigerator monitoring Child and adolescent health: services, guidelines, training, equipment NCDs: cardiovascular and chronic respiratory care, training, equipment Surgery: services, equipment, oxygen supply, medicines and materials, documents and guidelines, training, infection control equipment Blood transfusion: availability and safety procedures Sickle cell disease: services, guidelines, trainings
	Section 6	Diagnostics	Availability of laboratory and imaging services, diagnostic equipment
	Section 7	Medicines and commodities	Availability of tracer medicines, vaccines, medical supplies
	Section 8	Interviewer’s observation	Facility functionality, cleanliness and service readiness verification

IP, inpatient; NCDs, non-communicable diseases; OP, outpatient; SARA, Service Availability and Readiness Assessment.

#### Phase II: implementation and data collection

In Phase II, the adapted toolkit will be implemented across all selected facilities. Each facility will be geo-mapped to assess accessibility and to support logistical planning. Preliminary engagement meetings will be held with facility heads to introduce the study’s objectives and procedures and to facilitate their support. Each facility will designate a focal staff member to coordinate the assessment.

Data collection will employ a combination of structured facility surveys, semistructured KIIs and observational checklists. The facility survey will capture quantitative information on infrastructure, human resources, equipment availability, laboratory capacity, essential medicines and service-readiness indicators. Observational checklists will be used to validate the availability and functional status of diagnostic tools, medicines and equipment.

Trained field investigators will undertake all data collection activities. Regular supervisory checks will be conducted to ensure the quality and completeness of the data, including the reliability substudy described above. Facility-based quantitative data collection is planned over a 6-month period in 2026 (see the Study design, setting and timeline section), during which each facility will require approximately 2–3 days to complete the assessment.

KIIs will be conducted following completion of quantitative data collection and preliminary analysis, to allow emerging quantitative patterns to inform interview probes. KIIs will be conducted with approximately 15–20 purposively selected participants, sampled to achieve maximum variation across facility level (primary, secondary, tertiary), professional cadre (medical officers, laboratory personnel, nursing staff and programme or data staff) and taluk. Recruitment will proceed iteratively, with the final number of interviews guided by the principle of information power and thematic saturation: data collection will continue until two consecutive interviews yield no substantively new themes relevant to the research questions. A structured interview guide has been developed to explore perceptions of health-system readiness, with key domains presented in table 4.

**Table 4:** Key interview domains and guiding questions used in key informant interviews and validation workshops

Section	Subthemes/guiding questions
I. Background	1. Role and experience Professional role within the health system or tribal health governance. Years of experience in public health, SCD management, laboratory systems, programme administration or tribal health. Prior involvement in NSCAEM, SCD screening or related health programmes.
II. Understanding of SCD and health system readiness	1. Perspectives on SCD burden and service needs Understanding of SCD prevalence, community challenges and care-seeking behaviours. 2. Facility and system readiness Perceptions of diagnostic, treatment and referral readiness in the district. Bottlenecks in infrastructure, HR, diagnostics, essential medicines (eg, hydroxyurea). 3. Validation of quantitative findings Presentation of quantitative readiness scores for validation. Expert reflections on whether the findings reflect ground realities. Explanations for observed gaps or unexpected patterns in the data.
III. Expert knowledge and programme implementation insights	1. Specific expertise and lived experience Insights based on clinical, managerial, laboratory or tribal health experience. Operational challenges faced during SCD screening, diagnosis or follow-up. 2. Policies, guidelines and programme execution Awareness of NSCAEM guidelines and alignment with district-level realities. Experience with implementation of related programmes (eg, NCD clinics, maternal and child health services, blood transfusion services). Resource gaps and policy barriers affecting implementation.
IV. Community and collaborative efforts	1. Community engagement and tribal participation Role of tribal leaders, Sanghas and community-based organisations in SCD care. Community trust in the health system and cultural considerations. 2. Intersectoral collaboration Partnerships between health facilities, NGOs, academic institutions and district health authorities. Scope for strengthening collaboration for SCD screening, follow-up and counselling.
V. Future directions	1. Vision and recommendations for strengthening readiness Expert views on priority interventions needed to improve readiness. Suggestions for immediate, medium-term and long-term actions (aligned with your Phase III outputs). Opportunities for integrating SCD services into existing programmes and systems strengthening initiatives.

NACAEM, National Sickle Cell Anaemia Elimination Mission; NCDs, non-communicable diseases; NGO, Non-Governmental Organization; SCD, sickle cell disease.

All interviews will be audio-recorded with participant consent, transcribed verbatim and translated into English where necessary. Data will be managed and coded in NVivo (V.14) using a hybrid deductive-inductive framework: an initial coding framework will be derived from the WHO Health System Building Blocks, and the study’s a priori domains of interest (table 4), supplemented by inductive codes emerging from the data. Coding will be performed independently by two researchers, with discrepancies resolved through discussion and, where necessary, adjudication by a third team member; a coding manual will be maintained and iteratively refined to ensure consistency.

#### Phase III: refinement and stakeholder validation

In Phase III, preliminary findings from both the quantitative facility survey and the qualitative KIIs will be synthesised and presented in a single stakeholder validation workshop, conducted after both data streams have been analysed. The workshop will include approximately 20–25 participants, purposively selected to represent district health administration (eg, district health officer, district programme manager), facility-level providers drawn from a cross-section of participating facilities, academic and technical experts (including members of the toolkit-adaptation panel) and representatives of tribal community organisations and Sanghas active in SCD-affected areas.

The workshop will follow a structured format: preliminary quantitative and qualitative findings will be presented together, followed by a facilitated small-group discussion using a nominal group technique, in which participants rate and discuss the accuracy, completeness and contextual relevance of the findings, and jointly identify and prioritise implementation gaps into immediate, medium-term and long-term categories. Discussion outputs will be recorded, thematically synthesised by the research team, and used to (1) refine the readiness toolkit for future application, (2) contextualise quantitative and qualitative findings in the final manuscript and (3) co-develop actionable, context-specific recommendations for strengthening NSCAEM implementation, which will be shared with district and state health authorities.

## Data analysis

### Quantitative analysis

Quantitative data will be analysed using the WHO SARA analytical framework, adapted to include SCD-specific indicators. Data will be cleaned and checked for completeness, internal consistency and logical errors; discrepancies will be verified against source records.

### Construction of readiness indices

Following the WHO SARA analytical framework, readiness will be assessed quantitatively across the following domains: basic amenities, basic equipment, standard precautions for infection prevention, diagnostic capacity, essential medicines and service-specific readiness for SCD. Within each domain, tracer indicators will be coded as binary variables (1=available and functional; 0=unavailable or non-functional). Domain scores will be calculated as the proportion of tracer indicators available within each domain and expressed as percentages.

The overall Service Readiness Index (SRI) for each facility will be calculated as the unweighted mean of the domain scores. Equal weighting was adopted for two reasons. First, it follows the analytic convention set out in the WHO SARA reference manual, under which composite readiness indices are conventionally constructed as unweighted means of domain scores to preserve comparability with other SARA-based assessments.^26^ Second, in the absence of an empirically validated, SCD-specific weighting scheme, an equal-weighting approach avoids introducing unsubstantiated assumptions about the relative importance of individual domains. We recognise, however, that domains may plausibly differ in their contribution to SCD service delivery (eg, diagnostic capacity may be more critical than basic amenities for confirmatory testing). A prespecified sensitivity analysis will therefore compare facility rankings and composite scores under the equal-weighting approach with those under an alternative, expert-elicited weighting scheme derived from a brief pairwise ranking exercise administered to the same expert panel consulted during toolkit adaptation. Concordance between the two sets of rankings will be assessed using Spearman’s rank correlation coefficient; material divergence between the two schemes would indicate that the choice of weighting materially affects interpretation and will be reported and discussed as a limitation of the composite index.

A service-specific SCD readiness index will also be constructed, incorporating indicators for screening, confirmatory diagnostics, medicines, crisis management, counselling, referral and follow-up. The scoring approach will mirror that of the general SRI. Composite scores will be presented alongside individual tracer indicators to ensure transparency and interpretability.

### Handling missing data

Missing responses will be documented and reviewed. For composite indicators, missing tracer values will be excluded from both the numerator and denominator for that facility to minimise bias. Sensitivity analyses will be conducted comparing results under alternative assumptions (eg, coding missing values as ‘not available’).

### Sensitivity and validation analyses

To assess robustness, SRI scores will be compared across facility levels, and, as described above, alternative weighting schemes will be explored using Spearman’s rank correlation. Observational checklist data and documentary verification will be used to validate self-reported responses. The reliability substudy described in Phase I (independent re-assessment of three randomly selected facilities) will provide an indicative estimate of inter-rater agreement for tracer items (Gwet’s AC1, reported alongside Cohen’s kappa for comparability) and for domain scores (ICC) once analysed, acknowledging the imprecision inherent to this small subsample. Discrepancies between data sources will be analysed qualitatively during the stakeholder validation workshop.

### Qualitative analysis

Qualitative data from KIIs and validation workshops will be transcribed verbatim and analysed thematically using the coding approach described under Phase II. Coding will be iterative and conducted by at least two researchers, with discrepancies resolved through discussion and collaboration. Qualitative findings will be triangulated with quantitative results to aid the interpretation of readiness gaps and to contextualise implementation barriers.

### Risk of bias and mitigation strategies

Several potential sources of bias may affect this study, and predefined measures will be implemented to minimise them. Selection bias will be addressed by including all eligible public health facilities providing SCD services in the district, rather than sampling a subset. Within facilities, respondents will be purposively selected based on their direct involvement in service delivery, and replacements will occur only when unavoidable.

Information and reporting bias may arise from reliance on self-reported data. To mitigate this, self-reported items will be verified, where feasible, through documentary review (including registers, stock records and equipment logs) and direct observation. Items will be classified as ‘functional’ only when functionality is demonstrated or clearly documented by health staff. Observer bias will be reduced through standardised training of data collectors, piloting of data collection tools, ongoing supervision with periodic cross-checks by the primary investigator, and the independent, blinded re-assessment of a random subsample of three facilities described above. A detailed field manual will guide decision-making for ambiguous responses.

Social desirability bias may influence staff responses. Interviews and facility assessments will therefore be conducted in private settings, with explicit assurance that findings will not be used for performance appraisal and will be reported only in aggregate. Missing-data bias will be minimised through real-time completeness checks, followed by verification with facility records. For readiness indices, missing values will be handled using predefined analytic rules, and sensitivity analyses will be conducted to assess the robustness of estimates. Finally, analytical bias will be reduced by using predefined scoring algorithms based on the WHO SARA framework. Quantitative findings will be triangulated with qualitative data and discussed during stakeholder validation workshops to support contextual interpretation and identify inconsistencies.

## Reporting standards and protocol transparency

This protocol is reported in accordance with the Strengthening the Reporting of Observational Studies in Epidemiology (STROBE) statement, adapted for cross-sectional studies,^32^ and follows the Good Reporting of a Mixed Methods Study (GRAMMS) recommendations for describing the rationale, design and integration of quantitative and qualitative components.^33,34^

The development and reporting of the service-readiness assessment are informed by WHO recommendations for implementation research and for health facility surveys. The study protocol was developed before data collection and has not undergone substantive changes since ethics approval. Any protocol amendments will be documented and reported transparently in subsequent publications arising from this work. The use of a standardised, publicly available framework (WHO SARA) supports methodological transparency and reproducibility.

## Ethical considerations

Ethical approval for this study has been obtained from the Institutional Ethics Committee of JSS Academy of Higher Education and Research, Mysuru, Karnataka (Reference: JSS/MC/PG/217/2024-25). All procedures will be conducted in accordance with the Declaration of Helsinki and applicable national research guidelines. Written informed consent will be obtained from all participants before data collection commences. Participants will receive clear information regarding the purpose of the study, the voluntary nature of participation, procedures involved, potential risks and benefits and their right to withdraw at any time without consequence. Separate consent will be obtained for the audio recording of interviews. Confidentiality will be strictly maintained. No identifiable information will be included in transcripts, databases or publications. Data will be anonymised, stored on password-protected devices and accessed only by authorised research team members. All records will be retained securely for the period required by institutional policy and then destroyed.

As this study does not involve clinical interventions or patient records, the risk to participants is considered minimal. Nevertheless, appropriate safeguards will be in place to protect privacy, prevent coercion and ensure respectful engagement with participants and communities.

## Dissemination plan

The dissemination of study findings will follow a multi-layered strategy designed to ensure accessibility, utility and policy uptake at multiple levels of the health system. The primary results will be shared with the Karnataka State Health Department and the District Health Office of Chamarajanagar through detailed policy briefs, supporting evidence-informed planning and implementation under NSCAEM. Findings will also be communicated through presentations at national and international public health conferences, enabling wider academic and policy engagement.

At the community level, results will be disseminated through tribal health forums, local Sanghas and collaborative meetings with networks working in tribal and rural health. Peer-reviewed publications in open-access public health journals will ensure that the findings contribute to the global evidence base on health-system readiness for SCD. Additionally, knowledge translation workshops will be conducted with Centre for Training, Research and Innovation in Tribal Health (CTRITH) partners, public health researchers and local decision-makers to facilitate the practical application of the study results in programme strengthening and future research.

## Discussion

This protocol outlines a systematic assessment of health-service readiness to deliver SCD-related services in Chamarajanagar District, aligned with the objectives of NSCAEM. NSCAEM represents a significant policy initiative in India, marking the first national mission focused on a genetic disorder and reflecting a shift towards addressing long-standing health inequities affecting tribal populations.^11,12,27^ However, the success of such an ambitious programme depends not only on expanded screening but also on the capacity of health facilities to provide timely diagnosis, clinical management, referral and long-term care. This protocol addresses the need for district-level evidence on service readiness to support the effective implementation of NSCAEM in high-priority settings.

Globally, SCD is recognised as a substantial but under-addressed public health burden, contributing significantly to preventable morbidity and mortality, particularly among children and marginalised populations.^1–5,13–15^ In India, the burden of SCD is concentrated among scheduled tribe communities, where geographic remoteness, socioeconomic disadvantage and health-system constraints intersect to limit access to comprehensive care.^6–10^ Although recent policy attention and large-scale screening efforts under NSCAEM have increased case detection,^12^ evidence suggests that gaps in diagnostic capacity, trained workforce, emergency preparedness and referral systems persist, especially in rural and tribal areas.^16–18^ Assessing the readiness of health facilities is therefore essential to ensure that screening translates into meaningful health gains.

Health-service readiness is widely recognised as a prerequisite for successful implementation of disease-control programmes in resource-constrained settings. The WHO SARA framework has been extensively used to evaluate facility capacity across various health conditions, including maternal and child health, HIV, malaria and non-communicable diseases.^20–22,24–26^ These studies have demonstrated the value of SARA in systematically identifying gaps in infrastructure, human resources, diagnostics and essential medicines, and in informing health-system planning and policy prioritisation. However, to date, no studies have applied the SARA framework to assess readiness for SCD services in India, particularly within tribal districts. This study addresses that gap by adapting the SARA tool to incorporate programme-specific and disease-specific indicators relevant to SCD care under NSCAEM, through a documented process of expert consultation and field piloting (see Phase I).

The proposed mixed-methods design strengthens the assessment by integrating quantitative facility-level indicators with qualitative insights from healthcare providers and programme stakeholders. Quantitative readiness scores enable a structured comparison across facility levels and service domains, while qualitative methods allow exploration of contextual, operational and sociocultural factors that may influence readiness but are not fully captured by structured surveys alone.^34,35^ Stakeholder validation workshops further enhance the credibility and applicability of findings by situating readiness scores within local implementation realities and facilitating dialogue on feasible solutions.

It is important to distinguish the scope of this assessment from adjacent, but conceptually distinct, questions. First, this study evaluates facility-level and provider-level readiness; it does not assess community-level determinants of care-seeking. Patient-level and community-level factors—including stigma, disease awareness, cultural beliefs about anaemia and genetic conditions and geographic or economic barriers to accessing care—are known to shape uptake of screening and treatment in tribal populations, but fall outside the scope of the present facility-readiness assessment. We regard this as a deliberate boundary of the study rather than an oversight; demand-side barriers require dedicated community-based research (eg, using validated stigma scales, health-belief-model-framed surveys or ethnographic approaches) and are identified below as a priority for future work.

Second, the exclusion of private and not-for-profit facilities reflects the fact that NSCAEM is implemented as a public-sector programme delivered through the National Health Mission architecture; private facilities are not formally integrated into the Mission’s screening, reporting or referral structure and do not receive the programme-specific inputs (eg, subsidised hydroxyurea, screening kits) evaluated in this assessment. We nonetheless recognise that private and not-for-profit providers deliver a substantial share of outpatient care nationally in India,^36^ and their exclusion may limit the generalisability of the readiness findings to the district’s overall health-seeking landscape. Therefore, the findings should be interpreted as reflecting the implementation of the government-led mission rather than the broader district healthcare ecosystem.

Third, as a single-district assessment, readiness profiles here are context-specific and should not be assumed to generalise directly to other NSCAEM districts. Chamarajanagar was purposively selected for its documented, comparatively high SCD burden among scheduled tribe populations,^23,36^ including the Soliga community, whose heterogeneous settlement patterns and varying contact with formal healthcare offer a demographically diverse patient base relevant to NSCAEM’s target population. The district’s remote, forested terrain and resource-constrained facility network impose genuine accessibility constraints on screening and referral—representative of NSCAEM’s core operational challenges rather than a best-case setting—and are broadly comparable to other high-priority tribal districts in NSCAEM’s initial rollout.^37^ The study also forms part of ongoing work under the DBT/Wellcome Trust India Alliance-funded CTRITH, based in Chamarajanagar. We therefore present this protocol as a replicable methodological framework rather than a source of nationally generalisable estimates, recommending replication in other high-burden tribal districts.

Fourth, and most fundamentally, readiness is a necessary but not sufficient precondition for effective service delivery. A facility may be classified as ‘ready’ by SARA criteria yet still deliver inconsistent or low-quality care because of provider practice variation, patient adherence or health-seeking behaviour. Readiness indicators primarily reflect the availability and functionality of inputs (structure) rather than the quality of care, service utilisation, treatment uptake or clinical outcomes (process and outcome).^19^ Improvements in NSCAEM screening coverage or facility readiness indices should therefore not be equated with improvements in morbidity, mortality or quality of life among people living with SCD. Longitudinal studies linking readiness indices to service utilisation, treatment continuity and clinical outcomes are required to establish this pathway empirically and are identified below as a priority for future research.

Several further methodological considerations are relevant to interpreting the anticipated findings of this study. As a cross-sectional assessment, the study will provide a snapshot of readiness at a single point in time and will not capture change over time. Although verification through observation, document review and the inter-rater reliability check described above is intended to mitigate reporting bias, some reliance on self-reported data remains. These limitations are inherent to facility-based readiness assessments and should be considered when interpreting the results.

Despite these limitations, the study is expected to generate policy-relevant evidence to support programme planning and health-system strengthening under NSCAEM. District-level readiness profiles can inform targeted investments in diagnostics, workforce training, essential medicines and referral systems, particularly in underserved tribal areas such as Chamarajanagar.^23,36^ By identifying specific readiness gaps, the findings can support more efficient resource allocation and the refinement of implementation strategies at the district and state levels. As highlighted by Piel and colleagues, the success of NSCAEM depends not only on large-scale screening but also on the readiness of health facilities to provide confirmatory testing, treatment and follow-up care. Systematically identifying readiness gaps can enable more efficient resource allocation and the refinement of implementation strategies at the district and state levels.^38^ The methodological approach described in this protocol can also be adapted for use in other districts and states implementing NSCAEM, thereby contributing to more systematic monitoring of readiness for SCD services nationwide.

Future research should build on this readiness assessment in three principal directions: (1) longitudinal studies tracking how improvements in facility readiness translate into service quality, continuity of care and clinical outcomes among people living with SCD; (2) community-based research exploring awareness, cultural perceptions of SCD, stigma and health-seeking behaviour among tribal populations, to complement the facility-level focus of the present study and (3) extension of the adapted SARA framework to private and not-for-profit providers, to build a more complete picture of the SCD care landscape. Together, these directions will support a more comprehensive, equity-oriented evidence base for SCD care in India.

## Strengths and limitations

This protocol employs a structured, standardised approach to assessing health-system readiness, using an adapted WHO SARA framework in conjunction with SCD-specific indicators aligned with NSCAEM. The adapted toolkit was informed by expert consultation and refined through field piloting, and measurement consistency was further examined through an independent, blinded re-assessment of a random subsample of three facilities during Phase II. Inclusion of all eligible public health facilities in the district enhances representativeness and supports district-wide interpretation. The mixed-methods design, incorporating facility surveys, observations and qualitative validation, enables triangulation and strengthens contextual understanding of readiness gaps.

Several limitations are acknowledged. The toolkit-adaptation consultation was a single-round, qualitative expert review rather than a structured Delphi process, and no formal content validity index was computed; this is a methodological limitation common to many context-adapted readiness tools. The cross-sectional design captures readiness at a single point in time and does not allow assessment of temporal change. Reliance on self-reported data introduces potential reporting bias, which is mitigated but not eliminated by observation, document review and the independent reliability check described above. That check was limited to four facilities, so the resulting agreement estimates will carry wide CIs and should be interpreted as indicative rather than definitive. Readiness indicators provide information on capacity rather than on the quality of care or patient outcomes, and findings are specific to a single tribal district and should not be generalised to other settings without caution. The exclusion of private-sector facilities and of community/patient perspectives narrows the scope of inference, as discussed above. Finally, adaptations to the SARA framework, while necessary for SCD, may limit direct comparability with other SARA-based surveys.

## Supplementary material

10.1136/bmjph-2026-005317 online supplemental file 1

## Data Availability

Data sharing not applicable as no datasets generated and/or analysed for this study.
