# Supplementary material for: Health service readiness for implementing the National Sickle Cell Anaemia Elimination Mission in Chamarajanagar District, Karnataka, India: a cross-sectional mixed-methods protocol
Source: BMJ Public Health. 2026 Sep 17;4(3):e005317. doi: 10.1136/bmjph-2026-005317 (PMC13599954; doi:10.1136/bmjph-2026-005317)
Supplement: Supplementary file 1 [file bmjph-4-3-s001.docx]

# **SUPPLEMENTARY MATERIAL**

## **Supplementary Table S1: SCD-specific tracer indicators used in the field toolkit, by domain**

| Domain | Tracer item |
| --- | --- |
| Staff (Trainings) and guidelines | National SCD guidelines for diagnosis and management available at facility (NSCAEM guideline document) |
|  | Provider(s) received SCD-specific training in the last two years |
|  | Provider(s) received training on the SCD application (ABHA/portal) for compatibility and use |
| Equipment | Blood pressure apparatus (digital or manual sphygmomanometer with stethoscope) |
|  | Weighing scale (adult / child / infant) |
|  | Light source (flashlight acceptable) |
|  | Pulse oximeter |
|  | Thermometer |
|  | Stethoscope |
|  | Refrigerator (functional cold-chain storage for vaccines and blood/blood products) |
| Diagnostics | Haemoglobin (Hb) |
|  | Renal Function Tests (RFT) |
|  | Solubility test |
|  | Sickling test |
|  | Peripheral Blood Film (smear) |
|  | Complete Blood Count (CBC) |
|  | Liver Function Tests (LFT) |
|  | Urine microscopy |
|  | Platelet count |
|  | Differential Leucocyte Count (DLC) |
|  | Reticulocyte count |
|  | Serum potassium |
|  | HPLC / Electrophoresis |
|  | MRI |
|  | ECHO / 2D-Echocardiogram |
|  | Blood Typing (ABO grouping) |
|  | Cross matching |
|  | ECG |
|  | X-Ray |
|  | TCD (Transcranial Doppler) |
| Medicine and commodities | Hydroxyurea Capsule |
|  | Clonidine Tablet |
|  | Ferrous Salt (A) + Folic Acid (B) |
|  | Ferrous Salts |
|  | Folic Acid Tablet |
|  | Iron Sucrose Injection |
|  | Heparin Sodium Injection |
|  | Warfarin Tablet |
|  | IFA syrup |
|  | NSAIDs |
|  | Ibuprofen tablet/syrup |
|  | Diclofenac tablet/injection |
|  | Paracetamol tablet/syrup |
|  | Penicillin |
|  | Clarithromycin Tablet |
|  | Desferrioxamine Injection |
|  | Enalapril Tablet |
|  | Tramadol capsule/injection |
|  | Morphine tablet/injection |
|  | Codeine tablet/oral solution |
|  | Hydroxyurea Tab |
|  | Tramadol Cap |
|  | Multivitamins |
|  | B12 Injections |
|  | Antiemetic (oral/injection) |
|  | Heparin |
|  | Diclofenac |
|  | Aceclofenac |
|  | Mefenamic acid |
|  | Iron and Folic Acid syrup |
|  | Morphine |
|  | Codeine |
|  | Desferrioxamine |
|  | Ibuprofen |
|  | Amitriptyline |
|  | Enalapril Tab |
